# Association between household size and risk of incident dementia in the UK Biobank study

**DOI:** 10.1038/s41598-024-61102-6

**Published:** 2024-05-14

**Authors:** Chao-Hua Cong, Pan-Long Li, Yuan Qiao, Yu-Na Li, Jun-Ting Yang, Lei Zhao, Xi-Rui Zhu, Shan Tian, Shan-Shan Cao, Jian-Ren Liu, Jing-Jing Su

**Affiliations:** 1 Department of Neurology, Shanghai Ninth People’s Hospital, Shanghai Jiao Tong University School of Medicine, No. 639 Zhizhaoju Road, Huangpu District, Shanghai, 200011 China; 2 Department of Medical Imaging, Henan Provincial People’s Hospital, Zhengzhou University People’s Hospital, No. 7 Weiwu Road, Zhengzhou, 450001 China; 3 School of Electrical and Information Engineering, Zhengzhou University of Light Industry, No. 5 Dongfeng Road, Zhengzhou, 450001 China; 4 Department of Neurology, Gongli Hospital of Shanghai Pudong New Area, No. 219 Miaopu Road, Pudong New District, Shanghai, 200135 China

**Keywords:** Household size, Dementia, Prospective cohort study, Brain structure, Neuroscience, Cognitive ageing, Cognitive neuroscience, Diseases of the nervous system

## Abstract

Currently, the relationship between household size and incident dementia, along with the underlying neurobiological mechanisms, remains unclear. This prospective cohort study was based on UK Biobank participants aged ≥ 50 years without a history of dementia. The linear and non-linear longitudinal association was assessed using Cox proportional hazards regression and restricted cubic spline models. Additionally, the potential mechanisms driven by brain structures were investigated by linear regression models. We included 275,629 participants (mean age at baseline 60.45 years [SD 5.39]). Over a mean follow-up of 9.5 years, 6031 individuals developed all-cause dementia. Multivariable analyses revealed that smaller household size was associated with an increased risk of all-cause dementia (HR, 1.06; 95% CI 1.02–1.09), vascular dementia (HR, 1.08; 95% CI 1.01–1.15), and non-Alzheimer’s disease non-vascular dementia (HR, 1.09; 95% CI 1.03–1.14). No significant association was observed for Alzheimer’s disease. Restricted cubic splines demonstrated a reversed J-shaped relationship between household size and all-cause and cause-specific dementia. Additionally, substantial associations existed between household size and brain structures. Our findings suggest that small household size is a risk factor for dementia. Additionally, brain structural differences related to household size support these associations. Household size may thus be a potential modifiable risk factor for dementia.

## Introduction

Dementia is a major and severe public health issue that greatly reduces the health-related quality of life, significantly increases the mortality rate, and poses a tremendous social and economic burden^1^. The dementia diagnosis rate in England was 64.6% in December 2023. By 2050, the global prevalence of dementia will soar to 150 million cases^2^. Given that modifiable risk factors accounting for approximately 40% of dementia cases, addressing these preventable risk factors has become a crucial priority in alleviating the global burden of dementia^2,3^.

Household size is increasingly recognized as a crucial social factor related to social networks, lifestyles, and socioeconomic characteristics^4,5^. Although previous research has reported an association between living alone and dementia^6,7^, the influence of household size on dementia incidence remains unclear. One cross-sectional study found that large household size was associated with a lower risk of dementia^8^. However, this study had limitations, including a small sample size, regional focus (limited to inhabitants of eastern Uttar Pradesh, India), and lack of long-term follow-up. To address this gap, a large population-based prospective cohort study is needed to examine whether household size is associated with the risk of dementia.

Moreover, little is known about the imaging biomarkers underlying the association of household size with dementia and preclinical changes in brain structure, which may reflect the cognitive benefits of living with large household size^9,10^. Previous studies have demonstrated the potential effects of living alone on the brain and white matter hyperintensity (WMH) volumes derived from magnetic resonance imaging (MRI)^11,12^. However, the associations of household size with total and regional brain volumes, WMH volumes, and white matter microstructure require further investigation. Recently, MRI modalities have been widely used to identify biomarkers for neurodegenerative diseases such as Alzheimer’s disease (AD) and age-related cognitive decline^10,13^. These pathology MRI biomarkers could be used to improve diagnostic sensitivity and accuracy, and might lead to novel molecular-based treatment interventions^14^. Therefore, identifying the factors that affect MRI indices related to cognitive function is crucial.

With this background, we aimed to explore the associations of household size with incident dementia after controlling for various confounders such as biological, social, and psychological factors. We utilized data from the extensive UK Biobank cohort. Additionally, we examined the impact of household size on neuroanatomical indices to uncover underlying mechanisms related to dementia risk.

## Methods

### Participants

The UK Biobank is a health-oriented, population-based prospective cohort study that recruited more than 500,000 participants aged between 40 and 69 years from 2006 to 2010 in the UK^15^. Ethical approvals of the UK Biobank were obtained from the National Information Governance Board for Health and Social Care and the North West Multi-Centre Research Ethics Committee^16^. All research was performed in accordance with the relevant guidelines and regulations. This study was approved under the UK Biobank application number 94885. For our analysis, we included participants who attended a baseline assessment center visit and excluded those with missing exposure variable data (specifically, responses such as “Do not know” or “Prefer not to answer” regarding household size) (*n* = 4549), a history of dementia (*n* = 283), without confounder data (*n* = 57,716), < 50 years old (*n* = 101,026), or without data of follow-up (*n* = 63,284). These exclusions resulted in a study sample of 275,629 participants. Neuro-imaging analyses were performed in 40,645 participants with quality-controlled MRI data and complete confounder information. The flowchart of the cohort and the study design is shown in Figure S1.

### Exposures

Information regarding household size was obtained from the Touchscreen questionnaire. Number in household was collected via a self-report question: “Including yourself, how many people are living together in your household? (Include those who usually live in the house such as students living away from home during term, partners in the armed forces or professions such as pilots)”. Responses falling below 1 or exceeding 100 were rejected. If a participant’s answer exceeded 12, they were prompted to confirm their response. In the primary analysis, we treated the number of individuals in the household as a continuous variable. In sensitivity analyses, we classified participants with the number of household = 1 as living alone (0) and those with the number of household > 1 as living with someone (1).

### Dementia diagnosis

The outcome of interest in this study was all-cause and cause-specific dementia, which were obtained from algorithmically (Category 47) defined outcomes in UK Biobank datasets. The International Classification of Diseases 10th revision (ICD-10) codes F00, F01, F02, F03, G30, G31, and ICD-9 were used to identify participants with incident dementia if any of these codes were recorded as a primary or secondary diagnosis in the health records. AD was identified by ICD-10 codes F00, G30, and ICD-9 code 290; vascular dementia (VD) was identified by ICD-10 code F01; and non-Alzheimer’s disease non-vascular (non-AD non-VD) dementia was identified by ICD-10 codes F02, F03, F05, F10, G31. Follow-up visits extended from the initial attendance at the assessment center until the earliest date of dementia diagnosis, death, or the last available date from the hospital inpatient data as of January 2022.

### Brain imaging data

For this study, the brain imaging information was obtained from three dedicated, identical imaging centers. Details of MRI acquisition protocol are available online^17^. All structural MRI data were preprocessed using a standard Siemens Skyra 3 T running VD13A SP4, with a standard Siemens 32-channel RF receive head coil, and quality-checked these data^18^. All images were subjected to nonlinear modulations and normalized for each individual head size^19,20^. Images were then smoothed with an 8 mm full width at half maximum Gaussian kernel with a resulting voxel size of 1.5 mm^3^. In this study, we focused our analyses on brain volume, WMH, white matter microstructure, cortical, and subcortical regions excluding the cerebellum.

### Covariates

The covariates for this analysis were selected based on previous literature and availability at baseline^21,22^. Sociodemographic factors included age, sex, ethnicity (White, Asian or Asian British, Black or Black British, other), educational level (≤ 10, 11–12, > 12 years), and socioeconomic status (SES). SES was measured by the Townsend deprivation index (TDI) representing area level deprivation categorized into 4 quintiles. Lifestyle factors were obtained by self-reported questions: smoking status (never, former, current smoking), alcohol intake (daily or almost daily, 3–4, 1–2 times/week, occasionally, never), and physical activity level (low, moderate, high). Biological factors comprised body mass index (BMI) (< 18.5, 18.5–24.9, 25.0–29.9, ≥ 30.0 kg/m^2^)^23^, APOE genotype (non-ε4-carrier, one-ε4-carrier, two-ε4-carrier)^24,25^, diabetes, stroke and high blood pressure. Depressed mood (not at all, several days, more than half the days, nearly every day) was tested using a self-reported question: “Over the past 2 weeks, how often have you felt down, depressed, or hopeless?”. Social isolation was quantified by three questions: number of people living together in the household (1 point if living alone), frequency of visits to or by friends or family (1 point if friends/family visits less than once a month), and engagement in leisure or social activities (1 point if no participation at least weekly)^15^. Participants with a total score of 2 or 3 were classified as with social isolation, while those with a total score of 0 or 1 were not. Detailed information on covariate definitions and collection is given in the appendix (Table 1).

**Table 1:** Outcomes and covariates definitions and method of assessment.

Covariate	Categorizations	UK Biobank code	Descriptions
Age (years)	Age in years	21,003	Age when attended assessment centre
Sex	Male, Female	31	NHS derived and/or touchscreen questionnaire
Ethnicity	White, Asian or Asian British, Black or Black British, Other	21,000	Touchscreen questionnaire:"What is your ethnic group?"
Education	Years of education ≤ 10, 11–12, > 12	6138,845	Touchscreen questionnaire:(i)"which of the following qualifications do you have?";(ii)"At what age did you complete your continuous full-time education?"
Socioeconomicstatus	Quartiles	22,189	Townsend deprivation index calculated prior to participant joining UK Biobank. Based on the preceding national census outputareas. Each participant is assigned a score corresponding to the output area in which their postcode is located
Smoking status	Never, previous, current	20,116	Touchscreen questionnaire:(i) "Do you smoke tobacco now?";(ii) "In the past, how often have you smoked tobacco?"
Alcohol intake	Daily or almost daily, 3–4 times/wk,1-2times/wk, Occasionlly, Never	1558	Touchscreen questionnaire:"About how often do you drink alcohol?"
Body massindex (kg/m^2^)	Under weight < 18.5, Normal(18.5,24.9), Over weight(25.0,29.9), Obese > = 30.0	21,001	Physical examination: body mass index
Physical activity level	Low, moderate, high	22,032,884	UK Biobank used International Physical Activity Questionnaire (IPAQ) to calculate the metabolic equivalent (MET) score. For those who were missing with MET score, we used the number of days per week they engaged immoderate physical activity to categorize their physical activity level
Hypertension	No, Yes	6150,20,002, 6177,6153	Touchscreen questionnaire and verbal interview:self-reported hypertension or anti-hypertensive medication use
Diabetes status	No, Yes	2443,20,002, 6177,6153	Touchscreen questionnaire and verbal interview:self-reported diabetes or insulin use
Prior stroke	No, Yes	6150,20,002	Touchscreen questionnaire and verbal interview:self-reported previous stroke
Depressive symptoms	Nearly every day, More than half the days, Several days, Not at all	2050	Touchscreen questionnaire:"Over the past two weeks, how often have you felt down, depressed or hopeless?"
APOE e4	No APOE e4, One APOEe4, Two APOEe4	rs7412,rs429358	Number of APOE e4: none (e2/e2, e2/e3 or e3/e3 haplotypes), One (e3/e4 or e2/e4 haplotypes), and two (e4/e4 haplotypes)
Social isolation	No(composite score < 2) Yes(compositescore ≥ 2)	709,1031,6160	Touchscreen questionnaire: (i)"Including yourself, how many people are living together in your our household?"(1 point if living alone);(ii)"How often do you visit friends or family or have them visit you?"(1 point if friends/family visits less than once a month)(iii)"Which of the following [leisure/social activities] do you engage in once a week or more often?"(1 point if no activities selected)
Number in household	Number in household	709	Touchscreen questionnaire:"Including yourself, how many people are living in your household (Include those who usually live in the house such as students living away from home during term, partners in the armed forces or professions such as pilots)?"

### Statistical analysis

#### Cox proportional hazard model

The Cox proportional hazards model was used to estimate the hazard ratios (HRs) and confidence intervals (CIs) for the relation of household size to incident dementia. The primary analyses were aimed to assess the relation of household size to all-cause dementia, followed by separate analyses with dementia subtypes of AD, VD, and non-AD non-VD dementia. We adjusted the models in several steps. Model 1 was adjusted for age. Model 2 was further adjusted for sex, ethnicity, APOE allele status, and education. Model 3 included Model 2 additionally adjusted for smoking status, alcohol intake, physical activity, BMI, and TDI. Model 4 was additionally adjusted for hypertension status, diabetes status, stroke history, and depressive symptoms, besides covariates in Model 3. Restricted cubic spline curves based on Cox analysis were used to investigate the potential of non-linearity, adjusted for covariates as in Model 4. In subgroup analysis, we examined the interaction between household size and different strata factors. We used the likelihood ratio test for multiplicative interaction analysis. If the *p*-value associated with the interaction term is < 0.05, we conclude that the interaction effect is significant. We performed stratified analyses to estimate potential modification effects according to age (≥ 65, < 65 years), TDI (Q1, Q2, Q3, Q4), smoking status (never, previous, current), and education level (≤ 10, 11–12, > 12 years). Several sensitivity analyses were conducted to analyze the robustness of our findings. Firstly, we additionally adjusted for social isolation in Model 4, since social isolation is a risk factor for dementia^21^. Secondly, we repeated the analyses among the participants without stroke history. Thirdly, we only included dementia events that occurred at least 5 years after baseline to minimize the influence of reverse causation as suggested by previous studies^26–28^. Finally, we classified participants as living alone and living with someone.

#### Brain imaging analysis

We employed linear regression models to explore the cross-sectional relationship between household size and brain morphometric measures. Our covariate adjustments followed the same approach as in Model 4. Two sensitivity analyses were performed: (1) classifying participants as living alone and living with someone; (2) excluding participants with nervous system disorders (e.g., dementia, stroke, brain surgery, multiple sclerosis, encephalitis, myelitis, encephalomyelitis, intracranial and intraspinal abscess and granuloma, acute disseminated demyelination and other demyelinating diseases of central nervous system) (*n* = 8,907). Owing to the number of statistical tests we performed, a Bonferroni correction for multiple testing was applied^29^. 21 independent brain MRI indices were tested. Therefore, the significance level *P* = 0.05 was divided by 21, which provides a significance level corrected for multiple testing: *P* = 0.0024^30^.

All statistical analyses were conducted using SPSS 23 and R (version 4.2.3). All P values listed below were adjusted.

### Informed consent

The participants of this study were from the UK biobank database. All participants gave informed consent through electronic signature before joining the UK biobank research project.

## Results

### Participant characteristics

Table 2 shows the baseline characteristics of the 275,629 dementia-free participants in the primary analyses. The mean (SD) age of participants was 60.45 (5.39) years and the average (SD) number in household was 2.20 (1.15). Women accounted for 52.63% of the sample. During a mean follow-up time of 9.50 years, 6,031 (2.19%) incidences developed all-cause dementia, including 2,565 cases of AD, 1,347 cases of VD, and 2,414 cases of non-AD non-VD dementia. At the imaging visit, 40,645 individuals without outliers were included, in which they had a mean (SD) age and household size of 55.97 (7.52) years and 2.55 (1.21), respectively. Women made up 52.43% of this subgroup.

**Table 2:** Baseline characteristics^a^ of the study population in the UK Biobank, stratified by incident dementia status.

Characteristic	Overall (N = 275,630)	Incident Dementia (N = 6,031)	No Incident Dementia (N = 269,599)
Mean follow-up duration, years	9.50	9.26	9.51
Age, years (Mean [SD])	60.45(5.39)	64.58(4.06)	60.35(5.38)
Sex, Female (%)	145.056(52.63)	2,785(46.18)	142,270(52.77)
Ethnicity (%)			
White	255,557(92.72)	5,602(92.89)	249,955(92.71)
Asian or Asian British	8,048(2.92)	130(2.16)	7,918(2.94)
Black or Black British	1,070(0.39)	21(0.35)	1,049(0.39)
Other	10,954(3.97)	278(4.61)	10,676(3.96)
Education, years (%) ≤ 10	74,617(27.07)	2,378(39,43)	72,239(26.80)
11–12	75,205(27.28)	1,536(25.47)	73,669(27.33)
> 12	125.807(45.64)	2,117(35.10)	123,690(45.88)
Socioeconomic status^b^ quintile (%)			
1 (least deprived)	73,377(26.62)	1,456(24.14)	71,921(26.68)
2	72,121(26.17)	1,472(24.41)	70,649(26.21)
3	68,691(24.92)	1,487(24.66)	67,204(24.93)
4 (most deprived)	61,440(22.29)	1,616(26.79)	59,824(22.19)
Smoking status (%)			
Never	141.571(51.36)	2,763(45.81)	138,808(51.49)
Previous	107,797(39.11)	2.670(44.27)	105,127(38.99)
Current	26,261(9.53)	598(9.92)	25,663(9.52)
Alcohol intake (%) Daily or almost daily	62,240(22.58)	1,302(21.59)	60,938(22.60)
3–4 times a week	63.973(23.21)	1,170(19.40)	62,803(23.30)
1–2 times a week	68,198(24.74)	1,361(22.57)	66,837(24.79)
Occasionally	59,697(21.66)	1,438(23.84)	58,259(21.61)
Never	21,521(7.81)	750(12.44)	20,761(7.70)
BMl,kg/m^2^ (%)			
< 18.5	1,270(0.46)	42(0.70)	1,228(0.46)
≥ 18.5 to < 25.0	83,138(30.16)	1,827(30.29)	81,311(30.16)
≥ 25.0 to < 30.0	120,871(43.85)	2,586(42.88)	118,285(43.87)
≥ 30.0	70,350(25.52)	1,576(26.13)	68,774(25.51)
Physical activity level (%)			
Low	57,550(20.88)	1,259(20.88)	56,291(20.88)
Moderate	113,188(41.07)	2,496(41.39)	110,692(41.06)
High	104.891(38.06)	2,276(37.74)	102,615(38.06)
Hypertension, yes (%)	96,035(34.84)	2,888(47.89)	93,147(34.55)
Diabetes status, yes (%)	17,638(6.40)	827(13.71)	16,811(6.24)
Prior stroke,yes (%)	99,041(35.93)	3,003(49.79)	96,038(35.62)
Depressed mood (%)			
Not at all	215.794(78.29)	4.601(76.29)	211.193(78.34)
Several days	47,589(17.27)	1,057(17.53)	46,532(17,26)
More than half the days	7,541(2.74)	229(3.80)	7,312(2.71)
Nearly every day	4.705(1.71)	144(2.39)	4.561(1.69)
APOE e4 (%)			
0	215,457(78.17)	3.464(57.44)	211,993(78.63)
1	54,759(19.88)	1.993(33.05)	52,766(19.57)
2	5,413(1.96)	574(9.52)	4,839(1.79)
Number in household (Mean [SD])	2.20(1.15)	1.96(0.97)	2.21(1.15)

BMI = body mass index, APOE = apolipoprotein E.

^a^Values are mean (SD) or *n* (%).

^b^Socioeconomic status measured using the Townsend deprivation index.

### Household size and dementia risk

In the unadjusted model, the HR for the incidence of all-cause dementia was 0.76 (95% CI, 0.74–0.79) for larger household size compared with small household size. However, after adjusting for various risk factors, this association was attenuated. The overall HR, accounting for all covariates, was 0.95 (95% CI, 0.92–0.98) (Table 3). The results for AD showed no significant association with household size in fully adjusted models. The results for VD and non-AD non-VD dementia showed consistent trends with those for all-cause dementia, that is, the risk of VD (HR, 0.93; 95% CI, 0.87–0.99) and non-AD non-VD dementia (HR, 0.92; 95% CI, 0.88–0.97) decreased with the increasing number of people in household in fully adjusted models (Table 3). The restricted cubic spline models revealed a reversed J-shaped association between household size and the risk of all-cause dementia, VD, and non-AD non-VD dementia. The lowest risk occurred at approximately 4 individuals in the household, after which the risk plateaued (Figure S2).

**Table 3:** Association between household size and risk of all-cause dementia, Alzheimer’s disease, vascular dementia, and non-Alzheimer’s disease non-vascular dementia.

	All-cause dementia	Alzheimer’s disease	Vascular dementia	Non-Alzheimer's disease
	(*N* = 6031)	(*N* = 2565)	(*N* = 1347)	Non-vascular dementia (*N* = 2414)
Number in household (SD)	1.96 (0.97)	1.97(1.06)	1.93(1.00)	1.96(0.93)
Person years	55,861	23,357	12,134	22,781
Unadjusted model	0.76(0.74,0.79;*p* < .001)	0.78(0.74,0.82;*p* < .001)	0.73(0.69,0.78;*p* < .001)	0.77(0.73,0.81;*p* < .001)
Model 1^a^	0.94(0.91,0.97;*p* < .001)	0.98(0.93,1.02;*p* = .271)	0.93(0.86,0.99;*p* = .031)	0.92(0.88,0.97;*p* = .002)
Model 2^b^	0.93(0.90,0.96;*p* < .001)	0.97(0.93,1.02;*p* = .267)	0.89(0.83,0.96;*p* = .003)	0.89(0.85,0.94;*p* < .001)
Model 3^c^	0.94(0.91,0.97;*p* < .001)	0.98(0.94,1.02;*p* = .371)	0.93(0.86,0.99;*p* = .027)	0.91(0.87,0.96;*p* < .001)
Model 4^d^	0.95(0.92,0.98;*p* < .001)	0.97(0.94,1.02;*p* = .265)	0.93(0.87,0.99;*p* = .032)	0.92(0.88,0.97;*p* = .001)

^a^Adjusted for age.

^b^Model 1 with additionally adjusted for sex, ethnicity, APOE allele status, and education.

^c^Model 2 with additionally adjusted for smoking status, alcohol intake, physical activity, BMI, and Townsend index of deprivation.

^d^Model 3 with additionally adjusted for the status of hypertension, diabetes, stroke history, and depressive symptoms.

**Figure 1:**
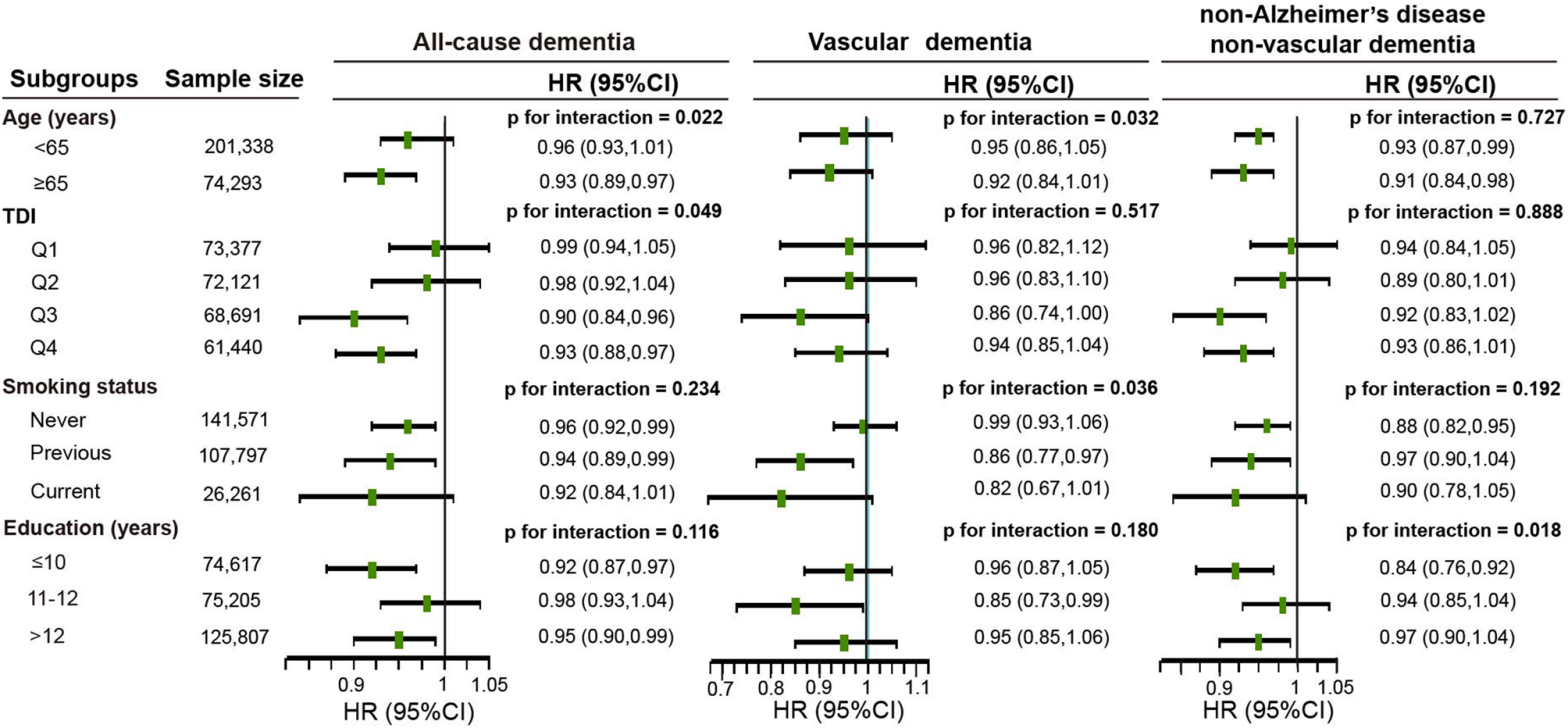
Associations of household size with all-cause and cause-specific dementia by age, TDI, smoking status, and education level. TDI, Townsend deprivation index; CI, confidence interval; HR, hazard ratio. In each model, potential confounders the same as Model 4 in Table 2 were adjusted.

We observed multiplicative interactions between household size and several factors in relation to the risk of all-cause dementia (Fig. 1). The association between household size and dementia risk was significant in older individuals (in ≥ 65 years: HR 0.93 [95% CI, 0.89–0.97]) and more TDI deprived groups (in Q3: HR 0.90 [95% CI, 0.84–0.96]; in Q4: HR 0.93 [95% CI, 0.88–0.97]). In the never smoking (HR, 0.96; 95% CI, 0.92–0.99) and previous smoking (HR, 0.94; 95% CI, 0.89–0.99) groups, the association was significant. Furthermore, the association was significant for individuals with in the education ≤ 10 years (HR, 0.92; 95% CI, 0.87–0.97) and > 12 years (HR, 0.95; 95% CI, 0.90–0.99). The associations of household size with VD and non-AD non-VD dementia did not appreciably vary between subgroups. In most subgroup analyses, there is sufficient statistical power to detect effects (Figure S3).

In sensitivity analysis, we excluded participants who had a history of stroke (*n* = 5,315) or those who developed dementia within 5 years (*n* = 536) of baseline assessment. Remarkably, the results remained consistent with the main analyses (Table S1 and Table S2). Additionally, when we further adjusted for social isolation in Model 4, the patterns of results remained similar (Table S3). After classifying participants as living alone and living with someone, the effects of living situation on dementia risk were even stronger (fully-adjusted HR 0.85; 95% CI 0.81–0.92) (Table S4).

### Associations of household size with brain structure

Whole-brain analyses revealed that total brain (grey and white) volume, total grey volume (GM), and the volumes of subcortical regions such as the thalamus, and accumbens were positively associated with household size (Table 4). WMH volume and white matter tract integrity indices (mean diffusivity [MD]) were negatively associated with household size. These findings remained robust when participants were classified as living alone or living with someone, and when patients with nervous system disorders were excluded.

**Table 4:** Association between household size and brain structure and volume.

Brain MRI indices analysis 2^b^	Number in household	Sensitivity analysis 1^a^	Sensitivity
	(*N* = 40,645)	(*N* = 40,645)	(*N* = 31,738)
Brain atrophy			
GM + WM	0.011(0.004,0.019;***p* = .002**)	0.057(0.035,0.080;***p* < .001**)	0.011(0.003,0.018;***p* = .007**)
WM	0.008(0.000,0.016;*p* = .055)	0.043(0.017,0.069;***p* = .001**)	0.007(− 0.002,0.016;*p* = .127)
GM	0.011(0.004,0.017;***p* = .001**)	0.051(0.030,0.071;***p* < .001**)	0.011(0.003,0.018;***p* = .004**)
WMH	0.013(− 0.021,− 0.006;***p* < .001**)	− 0.055(− 0.079,− 0.031;***p* < .001**)	0.014(− 0.022,0.006;***p* < .001**)
White matter tract integrity			
FA	0.010(0.001,0.018;***p* = .021**)	0.027(0.000,− 0.053;***p* = .048**)	0.008(− 0.001,0.018;*p* = .071)
MD	− 0.014(− 0.021,− 0.006;***p* < .001**)	0.041(− 0.066,− 0.016;***p* = .001**)	0.016(− 0.025,− 0.008;***p* < .001**)
ICVF	0.006(− 0.003,0.014;*p* = .180)	0.039(0.013,0.066;***p* = .003**)	0.004(− 0.005,0.014;*p* = .338)
ISOVF	− 0.010(− 0.018,− 0.002;***p* = .015**)	− 0.018(− 0.043,0.009;*p* = .188)	0.014(− 0.023,− 0.005;***p* = .003**)
Frontoparietal GM			
Frontal pole	0.010(0.003,0.018;***p* = .006**)	0.005(− 0.018,0.029;*p* = .652)	0.010(0.002,0.018;***p* = .017**)
Angular gyrus	0.009(0.001,0.018;***p* = .026**)	0.013(− 0.014,0.039;*p* = .346)	0.012(0.002,0.021;***p* = .013**)
Middle temporal gyrus (temporooccipital)	0.010(0.002,0.018;***p* = .017**)	− 0.011(− 0.038,0.014;*p* = .373)	0.011(0.002,0.020;***p* = .020**)
Superior frontal gyrus	0.004(− 0.004,0.012;*p* = .311)	− 0.009(− 0.035,0.017;*p* = .488)	0.004(− 0.005,0.013;*p* = .376)
Cingulate gyrus	− 0.003(− 0.008,0.007;*p* = .928)	0.023(− 0.049,0.003;*p* = .080)	0.000(− 0.008,0.009;*p* = .956)
Precuneous cortex	0.012(0.004,0.019;***p* = .004**)	0.017(− 0.008,0.042;*p* = .189)	0.013(0.004,0.021;***p* = .004**)
Subcortical volumes			
Hippocampus	0.007(− 0.001,0.015;*p* = .071)	0.001(− 0.025,0.026;*p* = .944)	0.006(− 0.002,0.015;*p* = .160)
Thalamus	0.016(0.008,0.023;***p* < .001**)	0.037 (0.013,0.060;***p* = .002**)	0.016(0.008,0.023;***p* < .001**)
Putamen	0.008(0.001,0.016;***p* = .030**)	0.018(− 0.023,0.024;*p* = .945)	0.009(0.001,0.017;***p* = .033**)
Pallidum	0.008(0.000,0.016;*p* = .060)	0.018(− 0.008,0.044;*p* = .167)	0.010(0.001,0.019;***p* = .029**)
Amygdala	0.000(− 0.009,0.008;*p* = .920)	− 0.010(− 0.036,0.016;*p* = .442)	0.002(− 0.007,0.011;*p* = .719)
Caudate	0.007(0.000,0.015;*p* = .053)	0.005(− 0.029,0.019;*p* = .673)	0.008(0.000,0.016;*p* = .057)
Accumbens	0.017(0.009,0.025;***p* < .001**)	0.047(0.023,0.072;***p* < .001**)	0.020(0.011,0.028;***p* < .001**)

*P* < 0.0024 was considered significant, as we had to correct our analysis for multiple testing (*P* < 0.0024 was calculated as: 0.05 divided by 21). Beta coefficients were from multiple linear models adjusted for covariates included in Model 4 of Table 2. GM, the volume of grey matter; WM, the volume of white matter; GM + WM, the volume of grey and white matter; WMH, white matter hyperintensity; FA, fractional anisotropy; MD, mean diffusivity; ICVF, intracellular volume fraction; ISOVF, isotropic volume fraction; MRI, magnetic resonance imaging.

^a^classifying participants as living alone and living with someone.

^b^participants with nervous system disorders were excluded.

Significant values < 0.05 are in bold.

## Discussion

In this 9.5-year follow-up study of 275,629 UK Biobank participants, we found that small household size was associated with a higher risk of all-cause dementia, VD, and non-AD non-VD dementia. Additionally, smaller household size correlated with increased WMH load and reduced GMVs in brain regions related to learning and memory, which might partly mediate the relationship between household size and cognitive function. These findings underscore the potential impact of household size on dementia risk.

To our knowledge, this is the first large-scale longitudinal cohort study controlling for various risk factors to investigate the associations between household size and dementia risk. Consistent with a previous cross-sectional study^8^, we found that small household size was associated with a 1.05-fold increased risk of developing dementia. Our results also revealed strong associations between household size and specific dementia subtypes: VD and non-AD non-VD. Notably, the risk reduction associated with larger household size was more pronounced for non-AD non-VD dementia than for VD. Interestingly, we observed a reverse J-shaped association between household size and dementia risk. Specifically, as the number of household members decreased, the risk of dementia increased. However, beyond a certain threshold (approximately 4 individuals), further increases in household size did not provide additional protection against dementia. This nuanced relationship highlights the complex interplay between social networks, socioeconomic status, household size, and cognitive health^31^. Consistent with previous population-based studies, our study reaffirms that living alone is a risk factor for dementia^32,33^.

We first identified multiple brain regions associated with household size in the largest sample to date. In the UK Biobank, participants with small household sizes had higher WMH volumes, reduced white matter tract integrity, and lower GMVs in several regions, including frontal pole, temporal cortex, angular gyrus, thalamus, precuneus cortex, putamen, and accumbens. Higher WMH volumes are a key predictive marker of cognitive decline progression^34,35^. Decreased FA and increased MD and ISOVF are associated with memory and executive dysfunction^36^. The frontal pole, temporal cortex, and other subcortical volume reductions are involved in cognitive functions, emotional processing, and social perception^37^.

The potential mechanisms linking household size with dementia may be elucidated by the following information. Living with others provides a persistent companionship and engagement, whereas living alone is a reliable proxy for social isolation^15,21^. Cognitive reserve theory suggests that lacking day-to-day companionship and social isolation may reduce mental stimulation and weaken neural connectivity, potentially leading to cognitive decline^38,39^. In addition, social isolation resulting from small household sizes may lead to stress responses, including chronic HPA axis hyperactivity and inflammation, both associated with an increased dementia risk^40^. Moreover, cognitive impairment often lead to increased social isolation, as individuals with declining cognitive abilities exhibit behavioral changes and a tendency to withdraw.

The following issues should be taken into account when interpreting our findings. Firstly, it’s worth noting that participants in the UK Biobank study were predominantly female, reported fewer health conditions, and tended to reside in less socioeconomically deprived areas compared to the broader population^41^. While previous research suggests that the lack of representativeness may not significantly affect the generalizability of exposure-mortality relationships^42^, the potential impact of selection bias on the associations between household size and dementia risk remains uncertain. Secondly, relying on participants’ health-related records to identify dementia cases in the UK Biobank study could introduce misclassification bias, particularly for early or mild dementia. Nevertheless, it’s important to note that this method demonstrated a relatively high positive predictive value (82.5%) for all-cause dementia^43^. Thirdly, the neuroimaging analyses included a population-based sample of participants, with only 10 cases of incident dementia. Consequently, the observed associations between household size, WMH, reduced white matter tract integrity, and GMVs were not directly linked to dementia incidence in our current study. Additionally, there is a time gap of 9.43 ± 2.01 years between the imaging data and baseline characteristics information for participants in the image analysis. Over time, changing confounding factors may influence the results. Fourthly, our study did not consider cultural factors that could impact household size and behavior^38^. Notably, individualistic cultures tend to exhibit a higher prevalence of individuals living alone and a lower frequency of marriage and childbearing. Fifthly, although plasma proteins can predict dementia risk up to 10 years in advance, the impact of dementia latent period on the associations between household size and dementia remains uncertain due to our lack of participant predictions^44^. Furthermore, among individuals who live alone or have limited support from relatives, the clinical manifestations of dementia may be more pronounced, especially if these relatives are of the same generation and have limitations in their caregiving functions. Therefore, the estimated prevalence of dementia within this population may be overestimated. Additionally, due to the limited availability of data on time-varying variables, we were unable to account for time-varying confounders in our study. This limitation is common in most cohort studies^22^. Moreover, if household size changed over time (with older adults having fewer family members)^45^, our observed association between household size and dementia risk might have been underestimated. Specifically, living alone could potentially be even more strongly linked to the outcome.

We revealed that small household size was associated with an increased risk of dementia, independent of various potential risk factors. By integrating neuroimaging data, we demonstrated that smaller household size was related to lower GMVs, and higher WMH, coupled with changes in white matter tract integrity. These structural differences might partly mediate the association between household size and dementia risk. Consequently, our results highlight the importance of interventions targeting small household sizes.

### Supplementary Information

Supplementary Information.

## Data Availability

https://www.ukbiobank.ac.uk.The datasets used and/or analysed during the current study available from the corresponding author on reasonable request.
