# Supplementary material for: Association between household size and risk of incident dementia in the UK Biobank study
Source: Sci Rep. 2024 May 14;14:11026. doi: 10.1038/s41598-024-61102-6 (PMC11094068; doi:10.1038/s41598-024-61102-6)
Supplement: Supplementary file 1 — Supplementary Information. [file 41598_2024_61102_MOESM1_ESM.pdf]

Association between household size and risk of incident dementia in the UK Biobank study

Chao-Hua Cong[1] | Pan-Long Li[2,3] | Yuan Qiao[1] | Yu-Na Li[1] | Jun-Ting Yang[1] | Lei Zhao[1] | Xi-Rui Zhu[3] | Shan Tian[1] | Shan-Shan Cao[4] | Jian-Ren Liu[1] | Jing-Jing Su[1]

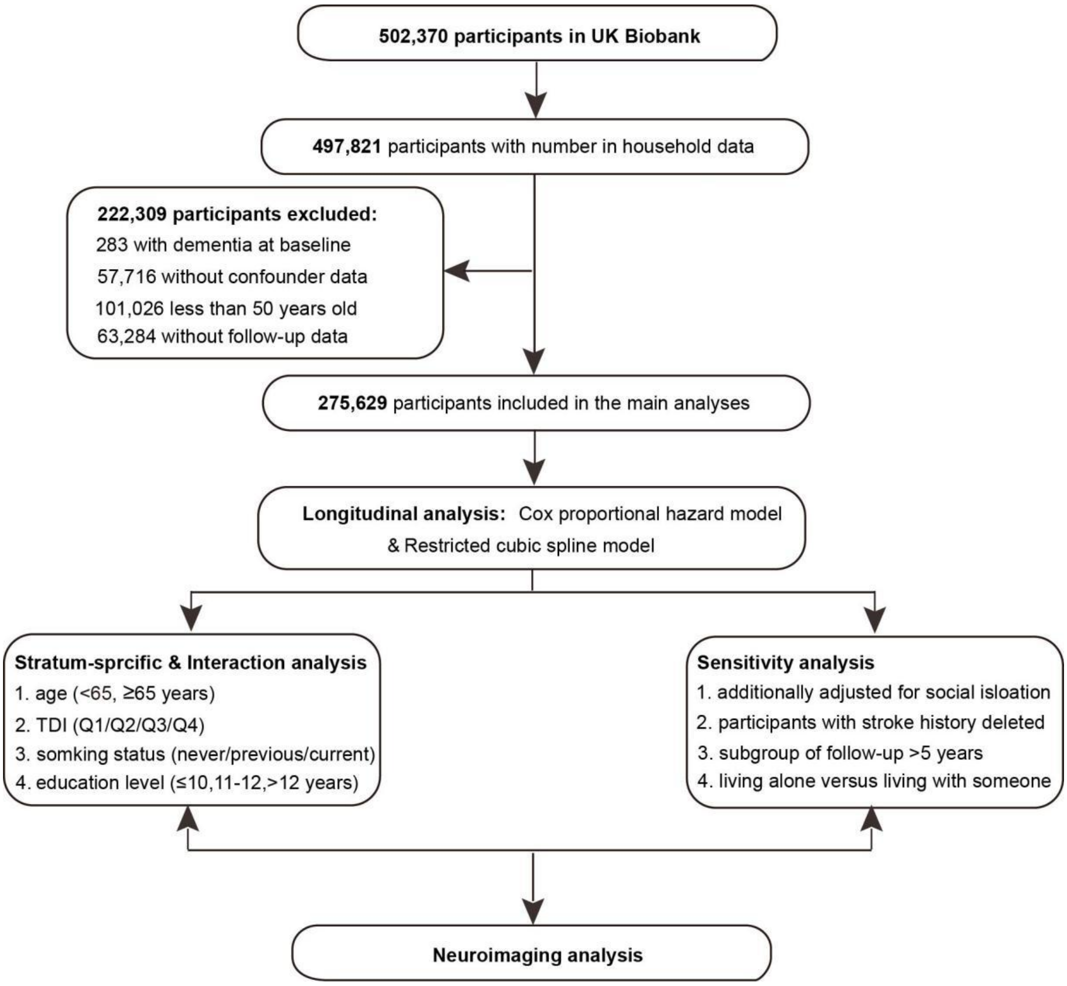

Figure S1 Flowchart of the study. Abbreviations: TDI, Townsend deprivation index.

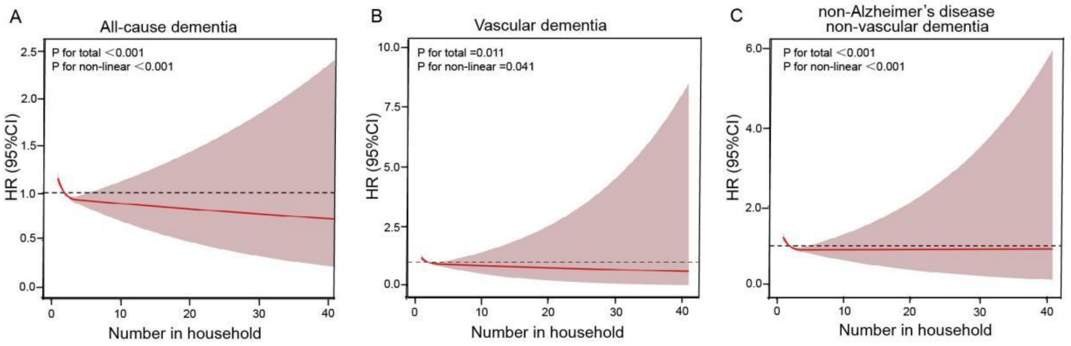

FIGURE S2 Nonlinear associations between household size and risk of all-cause dementia and cause-specific dementia. Restricted cubic spline fitted for Cox proportional hazards was used to flexibly model and visualize the relation of household size and risk of incident dementia, vascular dementia and non-Alzheimer's disease non-vascular dementia. Results were adjusted for potential confounders the same as Model 4 in Table 2. HR=hazar ratio.

Subgroups	Sample size	All-cause dementia		Vascular dementia		non-Alzheimer's disease non-vascular dementia	
		NO	power	NO	power	NO	power
Age (years)							
<65	201,338	2425	0.96	471	0.51	1079	1
≥65	74,293	3607	0.91	876	0.43	1335	0.88
TDI							
Q1	73,377	1456	0.16	304	0.87	585	0.78
Q2	72,121	1472	0.11	323	0.39	583	0.10
Q3	68,691	1487	1	313	0.88	604	0.90
Q4	61,440	1616	0.99	407	0.15	642	1
Smoking status							
Never	141,571	2763	1	564	0.12	1112	1
Previous	107,797	2670	0.71	620	1	1060	0.13
Current	26,261	598	1	163	0.99	242	1
Education (years)							
≤10	74,617	2376	1	579	0.15	878	1
11-12	75,205	1536	0.67	337	1	612	1
>12	125,807	2117	0.66	431	0.10	923	0.70

FIGURE S3 The information regarding sample sizes, dementia cases, and statistical power for all subgroups. NO， number of all-cause and cause-specific dementia.

	All-cause dementia (N=5,699)	Alzheimer's disaese (N=2,472)	Vascular dementia (N=1,193)	non-Alzheimer's disease non-vascular dementia (N=2,313)
Number in household (SD)	1.96 (0.98)	1.97 (1.07)	1.94 (1.02)	1,97 (0.93)
Person years	52,996	22,575	10,830	21,881
Unadjusted model	0.76 (0.74,0.79;p<.001)	0.77 (0.74,0.82;p<.001)	0.74 (0.69,0.79;p<.001)	0.77 (0.73,0.81;p<.001)
Model 1[a]	0.94 (0.91,0.97;p<.001)	0.97 (0.93,1.02;p=.259)	0.93 (0.87,1.01;p=.074)	0.93 (0.88,0.98;p=.005)
Model 2[b]	0.93 (0.90,0.96;p<.001)	0.97 (0.93,1.02;p=.280)	0.91 (0.84,0.98;p=.017)	0.90 (0.85,0.95;p<.001)
Model 3[c]	0.95 (0.92,0.98;p<.001)	0.98 (0.94,1.02;p=.387)	0.93 (0.87,1.01;p=.071)	0.92 (0.87,0.97;p=.001)
Model 4[d]	0.95 (0.92,0.98;p<.001)	0.98 (0.94,1.02;p=.342)	0.93 (0.88,0.97;p=.003)	0.90 (0.84,0.97;p=.006)

TABLE S1 Association of household size with dementia risk, excluding participants who had a history of stroke

[a] Adjusted for age.

[b] Model 1 with additionally adjusted for sex, ethnicity, APOE allele status, and education.

[c] Model 2 with additionally adjusted for smoking status, alcohol intake, physical activity, BMI, and Townsend index of deprivation.

[d] Model 3 with additionally adjusted for the status of hypertension, diabetes, stroke history, and depressive symptoms.

	All-cause dementia (N=5,494)	Alzheimer's disaese (N=2,335)	Vascular dementia (N=1,213)	non-Alzheimer's disease non-vascular dementia (N=2,205)
Number in household (SD)	1.95 (0.92)	1.96 (0.93)	1.92 (0.87)	1,96 (0.93)
Person years	54,071	22,551	11,680	22,136
Unadjusted model	0.76 (0.73,0.78;p<.001)	0.77 (0.73,0.81;p<.001)	0.72 (0.67,0.77;p<.001)	0.77 (0.73,0.81;p<.001)
Model 1[a]	0.94 (0.90,0.97;p<.001)	0.96 (0.91,1.01;p=.102)	0.90 (0.84,0.98;p=.015)	0.93 (0.88,0.98;p=.007)
Model 2[b]	0.92 (0.89,0.96;p<.001)	0.96 (0.91,1.01;p=.114)	0.88 (0.81,0.95; p=.001)	0.91 (0.86,0.96;p<.001)
Model 3[c]	0.94 (0.91,0.97;p<.001)	0.97 (0.92,1.01;p=.173)	0.91 (0.84,0.98;p=.0123)	0.92 (0.87,0.97;p=.003)
Model 4[d]	0.94 (0.91,0.97;p<.001)	0.97 (0.92,1.01;p=.132)	0.91 (0.85,0.98;p=.014)	0.93 (0.88,0.98;p=.005)

TABLE S2 Association of household size with dementia risk only including participants with dementia diagnosed at least five years after baseline

[a] Adjusted for age.

[b] Model 1 with additionally adjusted for sex, ethnicity, APOE allele status, and education.

[c] Model 2 with additionally adjusted for smoking status, alcohol intake, physical activity, BMI, and Townsend index of deprivation.

[d] Model 3 with additionally adjusted for the status of hypertension, diabetes, stroke history, and depressive symptoms.

	All-cause dementia (N=5,975)	Alzheimer's disaese (N=2,537)	Vascular dementia (N=1,339)	non-Alzheimer's disease non-vascular dementia (N=2,393)
Number in household (SD)	1.95 (0.98)	1.97 (1.06)	1.93 (1.00)	1,96 (0.93)
Person years	55,364	23,099	12,068	22,587
Unadjusted model	0.76 (0.74,0.78;p<.001)	0.78 (0.74,0.81;p<.001)	0.73 (0.68,0.78;p<.001)	0.77 (0.73,0.81;p<.001)
Model 1[a]	0.94 (0.91,0.97;p<.001)	0.97 (0.93,1.02;p=.213)	0.92 (0.86,0.99;p=.032)	0.92 (0.88,0.97;p=.002)
Model 2[b]	0.92 (0.89,0.95;p<.001)	0.97 (0.93,1.02;p=.214)	0.89 (0.83,0.96;p=.003)	0.90 (0.85,0.95;p<.001)
Model 3[c]	0.93 (0.88,0.97;p=.001)	0.96 (0.91,1.03;p=.273)	0.91 (0.82,1.00;p=.062)	0.89 (0.83,0.97;p=.005)
Model 4[d]	0.96 (0.93,0.99;p=.041)	1. 00 (0.96,1.05;p=.978)	0.95 (0.87,1.04;p=.261)	0.93 (0.87,0.99;p=.021)

TABLE S3 Association of household size with dementia risk, with the level of social isolation at baseline as a covariate

[a] Adjusted for age.

[b] Model 1 with additionally adjusted for sex, ethnicity, APOE allele status, and education.

[c] Model 2 with additionally adjusted for smoking status, alcohol intake, physical activity, BMI, and Townsend index of deprivation.

[d] Model 3 with additionally adjusted for the status of hypertension, diabetes, stroke history, and depressive symptoms.

	All-cause dementia (N=6,031)	Alzheimer's disaese (N=2,565)	Vascular dementia (N=1,347)	non-Alzheimer's disease non-vascular dementia (N=2,414)
live alone (%)	1547 (25.65)	613 (23.90)	362 (26.87)	642 (26.59)
Person years	55,861	23,357	12,134	22,781
Unadjusted model	0.72 (0.68,0.78;p<.001)	0.79 (0.72,0.87;p<.001)	0.68 (0.60,0.76;p<.001)	0.69 (0.63,0.76;p<.001)
Model 1[a]	0.79 (0.75,0.84;p<.001)	0.87 (0.80,0.96;p=.004)	0.75 (0.67,0.85;p<.001)	0.75 (0.68,0.81;p<.001)
Model 2[b]	0.78 (0.72,0.83;p<.001)	0.88 (0.81,0.96;p=.004)	0.71 (0.63,0.80;p<.001)	0.72 (0.65,0.79;p<.001)
Model 3[c]	0.83 (0.78,0.88;p<.001)	0.92 (0.85,1.02;p=.123)	0.80 (0.71,0.91;p<.001)	0.77 (0.69,0.85;p<.001)
Model 4[d]	0.85 (0.81,0.92;p<.001)	0.93 (0.85,1.03;p=.201)	0.83 (0.74,0.94;p=.005)	0.80 (0.72,0.88;p<.001)

TABLE S4 Association of household size with dementia risk, stratified by whether participants lived alone or with someone

[a] Adjusted for age.

[b] Model 1 with additionally adjusted for sex, ethnicity, APOE allele status, and education.

[c] Model 2 with additionally adjusted for smoking status, alcohol intake, physical activity, BMI, and Townsend index of deprivation.

[d] Model 3 with additionally adjusted for the status of hypertension, diabetes, stroke history, and depressive symptoms.
